# GSK3α and GSK3β Phosphorylate Arc and Regulate its Degradation

**DOI:** 10.3389/fnmol.2017.00192

**Published:** 2017-06-16

**Authors:** Agata Gozdz, Oleksii Nikolaienko, Malgorzata Urbanska, Iwona A. Cymerman, Ewa Sitkiewicz, Magdalena Blazejczyk, Michal Dadlez, Clive R. Bramham, Jacek Jaworski

**Affiliations:** ^1^Laboratory of Molecular and Cellular Neurobiology, International Institute of Molecular and Cell BiologyWarsaw, Poland; ^2^Department of Biomedicine and KG Jebsen Centre for Research on Neuropsychiatric Disorders, University of BergenBergen, Norway; ^3^Department of Neurology and Epileptology, Children’s Memorial Health InstituteWarsaw, Poland; ^4^Institute of Biochemistry and Biophysics, Polish Academy of ScienceWarsaw, Poland

**Keywords:** Arc/Arg3.1, GSK3, proteasomal degradation, phosphorylation, dendritic spines, neuronal activity, NMDA

## Abstract

The selective and neuronal activity-dependent degradation of synaptic proteins appears to be crucial for long-term synaptic plasticity. One such protein is activity-regulated cytoskeleton-associated protein (Arc), which regulates the synaptic content of α-amino-3-hydroxy-5-methyl-4-isoxazolepropionic acid receptors (AMPAR), excitatory synapse strength and dendritic spine morphology. The levels of Arc protein are tightly regulated, and its removal occurs via proteasome-mediated degradation that requires prior ubiquitination. Glycogen synthase kinases α and β (GSK3α, GSKβ; collectively named GSK3α/β) are serine-threonine kinases with abundant expression in the central nervous system. Both GSK3 isozymes are tonically active under basal conditions, but their activity is regulated by intra- and extracellular factors, intimately involved in neuronal activity. Similar to Arc, GSK3α and GSK3β contribute to synaptic plasticity and the structural plasticity of dendritic spines. The present study identified Arc as a GSK3α/β substrate and showed that GSKβ promotes Arc degradation under conditions that induce *de novo* Arc synthesis. We also found that GSK3α/β inhibition potentiated spine head thinning that was caused by the prolonged stimulation of N-methyl-D-aspartate receptors (NMDAR). Furthermore, overexpression of Arc mutants that were resistant to GSK3β-mediated phosphorylation or ubiquitination resulted in a stronger reduction of dendritic spine width than wildtype Arc overexpression. Thus, GSK3β terminates Arc expression and limits its effect on dendritic spine morphology. Taken together, the results identify GSK3α/β-catalyzed Arc phosphorylation and degradation as a novel mechanism for controlling the duration of Arc expression and function.

## Introduction

Activity-regulated cytoskeleton-associated protein (Arc; also known as Arg3.1) is a “short-lived” protein that is expressed in a neuronal activity-dependent manner. Arc emerged as a crucial regulator of various forms of synaptic plasticity (Bramham et al., 2010; Korb and Finkbeiner, 2011; Shepherd and Bear, 2011). Arc is required for the formation of long-term memory (Guzowski et al., 2000; Plath et al., 2006; Ploski et al., 2008) and maintenance of neuronal circuit homeostasis (Gao et al., 2010; Peebles et al., 2010). Arc appears to have multiple distinct roles in neuronal plasticity. First, the ability of Arc to organize the actin cytoskeleton was shown to be necessary for long-term potentiation (LTP) consolidation in hippocampal neurons (Messaoudi et al., 2007). Arc was also shown to regulate the strength of excitatory synapses through interactions with endocytic machinery elements, such as dynamin 2 and endophilins 2/3 (Chowdhury et al., 2006), stargazin (Zhang et al., 2015), and clathrin adaptor protein 2 (DaSilva et al., 2016), and promote endocytosis of the GluA1 subunit of α-amino- 3-hydroxy-5-methyl-4-isoxazolepropionic acid receptors (AMPARs). AMPAR endocytosis is required for homeostatic synaptic scaling (Shepherd et al., 2006) and metabotropic glutamate receptor (mGluR)-dependent long-term depression (LTD; Park et al., 2008; Waung et al., 2008; Jakkamsetti et al., 2013). Moreover, the Arc-dependent removal of AMPARs from the synapse influences dendritic spine morphology (Peebles et al., 2010). In addition to immediate actions of Arc at the synapse, Arc accumulates within the cell nucleus upon the massive induction of Arc synthesis and downregulates *GluA1* transcription (Korb et al., 2013).

Arc expression is induced by various stimuli, including glutamate (Lyford et al., 1995; Rao et al., 2006; Panja et al., 2009). The stimulation of glutamate N-methyl-D-aspartate receptors (NMDARs) is crucial for the induction of Arc expression during LTP at dentate gyrus synapses (Lyford et al., 1995; Panja et al., 2009). The increase in Arc in response to the γ-aminobutyric acid receptor (GABAR) antagonists bicuculline and picrotoxin requires NMDARs (Rao et al., 2006; Bateup et al., 2013). Similar to many other products of immediate early genes, Arc is both rapidly accumulated and quickly degraded by the ubiquitin-proteasome system (Rao et al., 2006; Greer et al., 2010; Soulé et al., 2012; Bateup et al., 2013; Mabb et al., 2014). To date, two E3 ubiquitin ligases, Triad3A and Ube3a, have been shown to tag Arc for subsequent proteasomal degradation (Greer et al., 2010; Mabb et al., 2014). Several proteins that are destined for proteasomal degradation need to be phosphorylated prior to tagging by E3 ubiquitin ligases, but the phosphorylation of Arc has not been investigated in this context.

Glycogen synthase kinases α and β (GSK3α and GSK3β; further collectively named GSK3α/β) are serine-threonine kinases with abundant expression in the central nervous system. Both are tonically active under basal conditions, but their activity changes in response to neurotrophic factors and neurotransmitters (Cole, 2012). GSK3α/β control both neuronal development and learning and memory processes (Salcedo-Tello et al., 2011; Cole, 2012), the latter of which occurs through an effect that is exerted by GSK3α/β on synaptic plasticity and the structural plasticity of dendritic spines. GSK3α/β activity is necessary for NMDAR-dependent LTD expression in hippocampal synapses (Peineau et al., 2007, 2009) and the maintenance of dendritic spine morphology under basal conditions (Ochs et al., 2015; Kondratiuk et al., 2017) or dendritic spine rearrangements upon chemical NMDAR-dependent LTD induction (Cymerman et al., 2015). GSK3α/β phosphorylate structural proteins that are crucial for synaptic plasticity, e.g., PSD-95 (Nelson et al., 2013), control actin dynamics within dendritic spines (Cymerman et al., 2015), regulate endocytosis at presynaptic (Clayton et al., 2010) and postsynaptic sites (Chen et al., 2007; Wei et al., 2010), and control the activity of the secreted protease matrix metalloproteinase-9 (Kondratiuk et al., 2017). Notably, in non-neuronal cells, many GSK3α/β substrates, upon their phosphorylation, undergo ubiquitination and proteasome-dependent degradation (Xu et al., 2009). To date, the most extensively studied protein that is degraded in a GSK3α/β-dependent manner is β-catenin. GSK3β/β-catenin pathway was shown to regulate excitatory transmission in hippocampal neurons under basal conditions *in vivo* (Ochs et al., 2015) and upon LTP induction in hippocampal slices (Chen et al., 2006). However, with the exception of β-catenin, the effect of the GSK3α/β-dependent degradation of synaptic proteins has not been thoroughly investigated.

In the present study, we identified Arc as a neuronal activity-related GSK3α/β substrate. Arc that was synthesized in response to extended NMDA treatment was subjected to phosphorylation by GSK3α/β and GSK3α/β-dependent degradation. At the same time prolonged NMDAR stimulation caused reduction of dendritic spine width. GSK3α/β inhibition enhanced the effect of NMDA on dendritic spine morphology, coinciding with an increase in Arc protein stability and expression. The overexpression of more stable, unphosphorylatable or ubiquitination-resistant mutants of Arc reproduced the effect of concomitant NMDAR stimulation and GSK3α/β inhibition on spine morphology, indicating that the GSK3α/β-dependent regulation of Arc turnover contributes to the structural plasticity of dendritic spines.

## Materials and Methods

### Animals

All of the procedures for harvesting animal tissue were approved by the First Ethical Committee (Warsaw, Poland; protocols no. 188/2011, 198/2011, 92/2015) and performed in accordance with the First Ethical Committee guidelines.

### Antibodies

The primary antibodies used in the study are listed in Table 1. Anti-phospho S170/T175 Arc antibody was developed by immunizing rabbits with KLH-conjugated phosphopeptide (GYDYTVS*PYAIT*P), and then cross-affinity purified using corresponding phosphorylated and non-phosphorylated peptides (Eurogentec, Liege, Belgium). Horseradish peroxidase (HRP)-conjugated secondary antibodies that were used for immunoblotting followed by enhanced chemiluminescence (ECL) detection were obtained from Jackson ImmunoResearch Laboratories (1:10,000; West Grove, PA, USA). IRDye 800CW-conjugated and IRDye 680LT-conjugated secondary antibodies for quantitative Western blot were obtained from LI-COR Biosciences (1:10,000; Lincoln, NE, USA). Alexa Fluor-conjugated secondary antibodies used for immunofluorescent staining were obtained from Thermo Fisher Scientific (1:200; Waltham, MA, USA).

**Table 1:** Antibodies.

Primary antibody (clone)	Manufacturer, catalog no.	Application, dilution
Rabbit anti-Arc (polyclonal)	Synaptic Systems (Goettingen, Germany), #156003	Western blot, 1:2000
		Immunocytochemistry, 1:300
Mouse anti-β-catenin (14/β-catenin)	BD Biosciences (Franklin Lakes, NJ, USA), #610154	Immunocytochemistry, 1:300
Mouse anti-GSK3α/β (21 A)	Thermo Fisher Scientific (Waltham, MA, USA), #44-610	Western blot, 1:2000
Mouse anti-α-tubulin (B 5-1-2)	Sigma-Aldrich (St. Louis, MO, USA), #T5168	Western blot, 1:20,000
Mouse anti-FLAG (M2)	Sigma-Aldrich, #F3165	Western blot, 1:1000
Rabbit anti-HA (C29F4)	Cell Signaling Technology (Danvers, MA, USA), #3724	Western blot, 1:1000
Rat anti-HA (3F10)	Sigma-Aldrich/Roche, #11867423001	Immunocytochemistry, 1:200
Mouse anti-GFP (C 163)	Thermo Fisher Scientific, #33-2600	Immunocytochemistry, 1:500

### Drugs

CHIR 98014 (CH98) was obtained from Axon Med Chem (Groningen, The Netherlands). BIO, anisomycin, cycloheximide, and NMDA were obtained from Sigma-Aldrich (St. Louis, MO, USA). MG-132 was obtained from Tocris Bioscience (Bristol, UK). NMDA and cycloheximide were dissolved in Neurobasal medium (Thermo Fisher Scientific) and methanol, respectively. The other drugs used in the study were dissolved in dimethylsulfoxide (DMSO).

### DNA Constructs

The following plasmids were described previously: β-actin-16pl (Kaech et al., 1996), pSuper (Brummelkamp, 2002), β-actin-GFP (Jaworski et al., 2005), β-actin-mCherry (de Vrij et al., 2008), BirA (de Boer et al., 2003), pEGFPC2-BIO (de Boer et al., 2003), BIO-βGal (Swiech et al., 2011), pGL4.11-SARE-ArcMin-luc2P (Kawashima et al., 2009), pUltra-Chili (gift from Dr. M. Moore, Addgene plasmid no. 48687), pCMV-VSV-G (Stewart et al., 2003), RSV-Rev (Dull et al., 1998), pMDLg/pRRE (Dull et al., 1998), HA GSK3β K85A pcDNA3, HA GSK3β S9A pcDNA3, and GSK3α pMT2 (all gifts from Dr. J. Woodget, Addgene plasmid no. 14755, 14754 and 15896, respectively). Genomic human and mouse Arc (IMAGE clones IRATp970A1095D and 3498057) were purchased from SourceBioScience (Nottingham, UK). GSK3α and β open reading frames were amplified by polymerase chain reaction (PCR) based on Addgene plasmid DNA and inserted into KpnI and NotI sites of a modified pcDNA3 FLAG vector, yielding GSK3α and GSK3β constructs that were FLAG-tagged on the C-terminus. A kinase dead GSK3α K148A and GSK3α S21A mutants were created by site-directed mutagenesis using Pfu Turbo DNA polymerase (Agilent, Santa Clara, CA, USA), based on wildtype GSK3α. To obtain efficient expression in neurons, FLAG GSK3β S9A was PCR-amplified and subcloned into BspTI and XbaI sites of the β-actin-16pl vector. pGEX Arc was generated by insertion of PCR-amplified Arc ORF using rat brain cDNA and the High Fidelity PCR enzyme mix (Thermo Fisher Scientific) into BamHI and XhoI sites of pGEX vector obtained from GE Healthcare Life Sciences (Buckinghamshire, UK). BIO-Arc constructs, which allow the expression of N-terminally avi-tagged Arc that is suitable for biotinylation, were generated by the insertion of PCR-amplified rat or human Arc ORFs into HindIII and EcoRI sites of modified pEGFPC2-BIO plasmid after EGFP sequence excision. Avi-tag contructs used for co-precipitation experiments were additionally HA-tagged on the N-terminus. SARE-Arc and SARE-mCherry constructs (Figure 3E) were generated in several steps. First, the Arc gene enhancer SARE and minimal Arc promoter (ArcMin) were PCR-amplified based on the pGL4.11-SARE-ArcMin-luc2P plasmid and inserted into XhoI and NotI sites of the pBluescript II KS (+) vector (Agilent), simultaneously generating a new SalI restriction site. Second, PCR-amplified genomic mouse Arc and mCherry sequences were PCR-cloned and ligated into SalI and NotI sites of a pBluescript plasmid that carried the SARE-ArcMin sequence. Third, BsrGI and BamHI sites were introduced by PCR into the genomic Arc sequence before the start codon. An HA oligonucleotide that was designed to have sticky ends that were compatible with BsrGI- and BamHI-generated ends was inserted, yielding an N-terminally tagged Arc construct. Lentiviral SARE-Arc vectors were generated by PCR cloning of the SARE-ArcMin-HA-Arc cassette, and insertion into PacI and NheI sites of pUltra-Chili remained after excision of the hUbC promoter and dTomato sequence. S170A/T175A, T368A/T380A, and S170A/T175A/T368A/T380A (4A) mutants were generated by PCR-based site-directed mutagenesis using BIO-Arc and SARE-Arc plasmids as a template. The same method was used to create the K136R mutant of SARE-Arc. To knockdown *Arc* expression, oligonucleotides encoding shRNAs targeting mouse *Arc* mRNA (later referred to as shArc) were inserted into BglII and HindIII sites of pSuper. The following sequences in *Arc* mRNA were targeted: ACCCAATGTGATCCTGCAG (Greer et al., 2010), GCGCTGGAAGAAGTCCATCAA and GGGTGGCTCTGAAGAATAT (both designed with the use of Whitehead Institute for Biomedical Research online tool)^1^.

**Figure 1:**
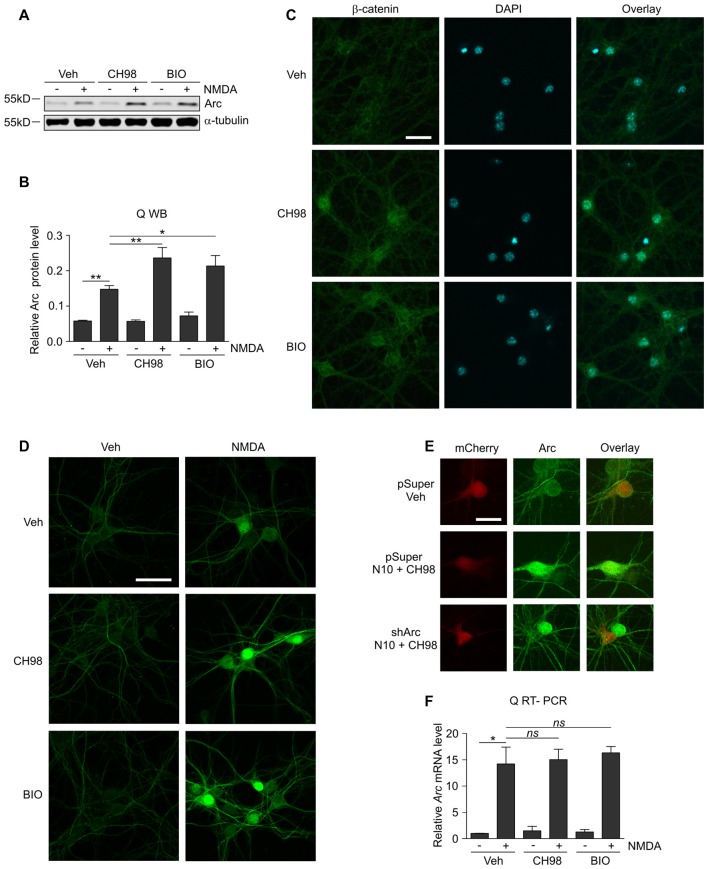
Glycogen synthase kinases α and β (GSK3α/β) inhibition increases Arc protein but not *Arc* mRNA levels in N-methyl-D-aspartate (NMDA)-stimulated neurons. **(A)** NMDA receptors (NMDAR)-dependent increase in Arc protein levels is augmented by GSK3α/β inhibition. Western blot analysis of Arc and α-tubulin expression in cortical neurons on day in vitro (DIV) 14–16 pretreated with vehicle, 1 μM CH98, or 1 μM BIO for 1 h and exposed for 4 h to 10 μM NMDA. **(B)** Quantification of Arc protein expression in neurons treated as in **(A)**, normalized to α-tubulin level. The data are expressed as mean ± standard error of the mean (SEM; *n* = 6 independent cultures). *p < 0.05, **p < 0.01 indicates statistical significance of the obtained results, estimated with one-way ANOVA with Bonferroni correction for multiple comparisons. **(C)** GSK3α/β inhibitors efficiently block β-catenin (i.e., the canonical target of GSK3α/β) degradation in neurons and promote its nuclear accumulation. Representative confocal images of β-catenin immunofluorescence in hippocampal neurons that were incubated with vehicle, 1 μM CH98, or 1 μM BIO for 4 h, fixed, immunolabeled for β-catenin, and stained with DAPI to visualize chromatin. Scale bar = 25 μm. **(D)** GSK3α/β inhibition promotes Arc accumulation in dendrites and nucleus of NMDA-stimulated neurons. The figure shows representative confocal images of Arc immunofluorescence in hippocampal neurons that were pretreated with vehicle or 1 μM CH98 or 1 μM BIO for 1 h and treated with 10 μM NMDA for 4 h. Scale bar = 50 μm. **(E)** Arc knockdown prevents NMDA and GSK3 inhibition-dependent increase in Arc immunofluorescence. The figure shows representative confocal images of Arc immunofluorescence in hippocampal neurons that were transfected on DIV12–13 with control vector (pSuper) or the mix of pSuper plasmids encoding shRNA against *Arc* mRNA together with β-actin-mCherry plasmid to visualize transfected cells. Two days postransfection cells were treated for 4 h as indicated. Scale bar = 25 μm.** (F)** GSK3α/β inhibition does not affect NMDAR-dependent *Arc* mRNA expression. The figure shows the results of the analysis of *Arc* mRNA levels in neurons that were stimulated with NMDA and GSK3α/β inhibitors. Cortical neurons were pretreated with GSK3α/β inhibitors, and Arc expression was induced with 10 μM NMDA for 2 h. *Arc* mRNA levels were determined by RT-qPCR, and the values were compared with controls (*Arc* mRNA levels in vehicle-treated cells). The data are expressed as mean ± SEM (*n* = 4 independent cultures). *p < 0.05 (one-way ANOVA with Bonferroni correction for multiple comparisons).

**Figure 2:**
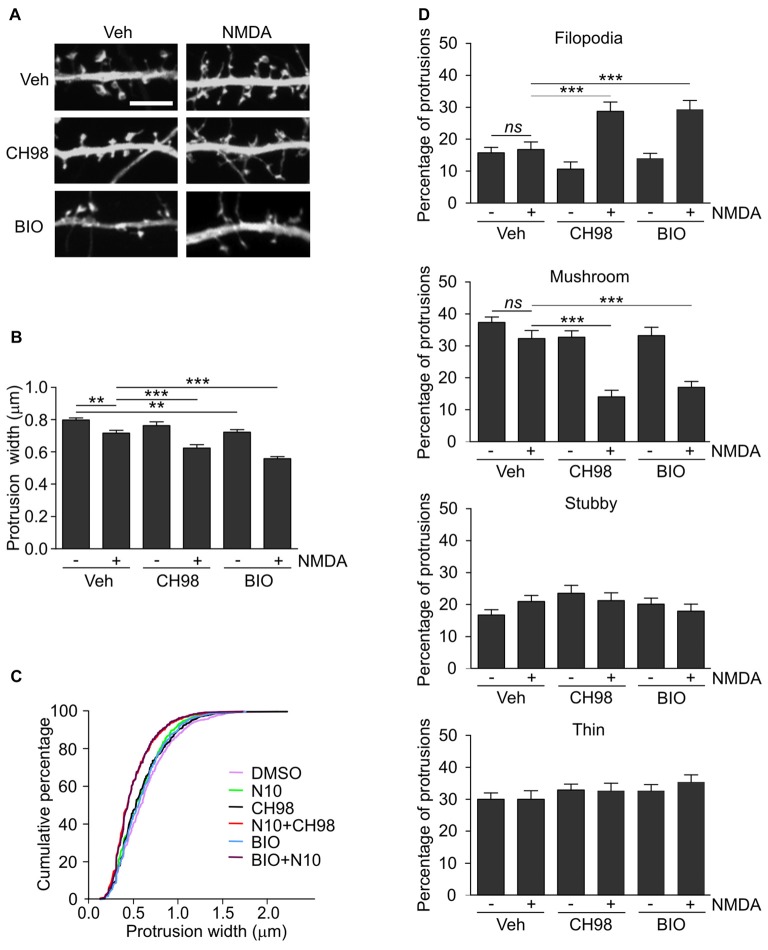
Combined treatment with NMDA and GSK3α/β inhibitors (CH98, BIO) leads to dendritic protrusion thinning and alterations in dendritic spine morphology. **(A)** Representative confocal images of dendritic protrusions. Hippocampal neurons were transfected with β-actin-GFP plasmid to visualize cell morphology and stimulated with 10 μM NMDA with or without 1 μM CH98 or 1 μM BIO for 4 h. Scale bar = 5 μm. **(B)** Quantitative analysis of dendritic spine width. Measurements were averaged per dendrite segment. Two dendrite segments per cell from 10 to 15 cells from two independent cultures were analyzed (5–8 cells per culture). The data are expressed as mean protrusion width ± SEM. **p < 0.01, ***p < 0.001 (one-way ANOVA with Bonferroni correction for multiple comparisons). **(C)** Cumulative percentage plot of protrusion width for neurons from **(B)**. Calculations were done for 522–680 spines per condition. **(D)** Analysis of dendritic protrusion categories for neurons from **(B)**. Categorization was done for dendrite segment and two dendrites per cell were analyzed. Data are expressed as mean percentage of all protrusions ± SEM. **p < 0.01, ***p < 0.001 (one-way ANOVA with Bonferroni correction for multiple comparisons).

**Figure 3:**
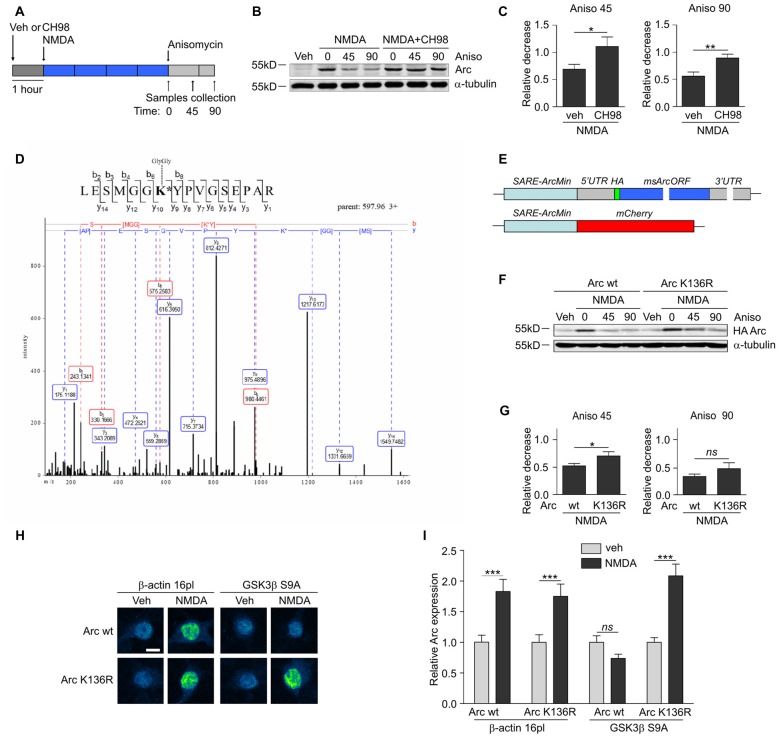
GSK3α and GSK3β promote Arc protein degradation and ubiquitination. **(A)** Schematic representation of cell treatment. Cortical neurons were pretreated with vehicle or 1 μM CH98 for 1 h, and then 10 μM NMDA was applied for 4 h. After this period, protein synthesis was inhibited with 40 μM anisomycin. The cells were harvested at the times indicated. **(B)** GSK3α/β inhibition results in slower Arc degradation. The rate of Arc degradation was evaluated by Western blot. **(C)** Quantification of Arc protein degradation rate in neurons treated as in **(A)**. Arc expression level was first normalized to α-tubulin level. Next, relative decrease in Arc expression was calculated as a ratio of Arc level at a given time point (45 or 90 min of anisomycin treatment) to Arc level at the “time 0”, when anisomycin was added to neurons pretreated with NMDA or NMDA with 1 μM CH98. Decrease in Arc levels in NMDA and NMDA with GSK3α/β inhibitor-treated neurons was compared separately for 45 or 90 min of anisomycin treatment and the difference was evaluated with *t*-test. The data are expressed as mean ± SEM (*n* = 6 independent cultures). *p < 0.05, **p < 0.01. **(D)** A novel GSK3β-dependent ubiquitination site is present in Arc protein. The figure shows representative fragmentation of a peptide that encompassed the ubiquitinated K136 residue, derived from rat Arc protein upon its co-expression with GSK3β S9A in HEK293 cells and incubation with the proteasome inhibitor MG-132 (10 μM) for 1.5 h. **(E)** Schematic representation of SARE-Arc and SARE-mCherry cassettes. **(F)** Lysine 136 ubiquitination is important for Arc degradation in neurons. The figure shows the Western blot analysis of the levels of HA-tagged Arc that was overexpressed in cortical neurons. Cells that were transduced with lentiviral vectors that carried wildtype SARE-Arc or SARE-Arc K136R were treated with vehicle or NMDA for 4 h. Anisomycin was then added to the neurons that were treated with NMDA for the times indicated. **(G)** Quantification of Western blot results from **(F)**. HA-tagged Arc expression levels were first normalized to α-tubulin levels. Next, relative decrease in HA-Arc expression was calculated as a ratio of HA-Arc level at a given time point (45 or 90 min of anisomycin treatment) to HA-Arc level at the “time 0”, when anisomycin was added to neurons pretreated with NMDA. Decrease in Arc and Arc K136R levels was compared separately for 45 or 90 min of anisomycin treatment and the difference was evaluated with *t*-test. The data are expressed as mean ± SEM (*n* = 5 independent cultures). *p < 0.05 (*t*-test). **(H)** K136R mutant of Arc is resistant to GSK3β-induced degradation. The figure shows representative confocal images of HA-tagged Arc expression in hippocampal neurons. Images were transformed to “blue to yellow” pseudo color mode to visualize differences in immunofluorescence intensity. Scale bar = 10 μm. Cells were co-transfected with β-actin 16pl vector or GSK3β S9A and wildtype SARE-Arc or the K136R mutant together with SARE-mCherry plasmid for 20 h and stimulated with NMDA for 4 h. **(I)** Quantitative analysis of HA-tagged Arc expression. For each cell, the HA immunofluorescence intensity was normalized to mCherry fluorescence intensity. For each kind of transfection, the expression of HA-tagged Arc in NMDA-stimulated cells was compared with its expression in unstimulated cells. Immunofluorescence intensity was measured from 60 to 70 cells per condition, derived from three independent cultures. ***p < 0.001; ns, not significant (Mann-Whitney test).

### Dissociated Neuronal Culture Preparation, Transfection, Lentiviral Transduction and Pharmacological Treatment

Dissociated hippocampal or cortical primary neuronal cultures were prepared from embryonic day 17 FVB mouse embryos. They were cultured as previously described (Cymerman et al., 2015) and used for experiments on day *in vitro* (DIV) 12–16. Hippocampal neurons were transfected on DIV 12–13 using Lipofectamine 2000 (Thermo Fisher Scientific) as described previously (Cymerman et al., 2015) and treated 48 h post transfection when shRNA was used. All other transfections were performed on DIV 13–14 and cells were treated 20 h later. To transduce cortical neurons with lentiviral vectors, cells on DIV 14–15 were incubated with lentiviral particles for 5 h followed by two washes in Neurobasal medium. Transduced neurons were used for further experiments 16 h later. NMDAR stimulation with 10 μM NMDA was performed in neuronal culture medium and lasted 4 h, as previously described (Bloomer et al., 2008). In experiments, where *Arc* mRNA expression was measured, incubation with 10 μM NMDA was shortened to 2 h. When applicable, cells were pretreated with vehicle (DMSO) or GSK3α/β inhibitors for 1 h.

### HEK293 and HEK293T Cell Line Cultures

HEK293 and HEK293T cells were purchased from ATCC (Manassas, VA, USA) and cultured in Dulbecco’s Modified Eagle Medium (DMEM) with 4500 mg/ml glucose supplemented with 10% fetal bovine serum (FBS) and penicillin-streptomycin (all from Sigma-Aldrich).

### Lentiviral Particle Production

To pack SARE-Arc DNA into lentiviral particles, HEK293T cells were transfected with lentiviral SARE-Arc vectors and packaging plasmids (pCMV-VSV-G, pRSV-Rev and pMDLg-pRRE) using the calcium phosphate transfection method. Six hours later, the transfection mix was removed. The cells were rinsed with phosphate-buffered saline (PBS), 137 mM NaCl, 2.7 mM KCl, 10 mM Na_2_HPO_4_, and 2 mM KH_2_PO_4_, pH 7.4, and grown in standard neuronal culture medium without glutamate for the next 40 h. The culture medium that contained viral particles was then centrifuged at 2000× *g* for 7 min at 4°C. The supernatant was aliquoted and stored at −80°C.

### RNA Isolation, Reverse Transcription and Quantitative Polymerase Chain Reaction

RNA isolation and cDNA synthesis were performed as described previously (Malik et al., 2013). Quantitative real-time PCR (RT-qPCR) was run on a 7900HT real-time PCR thermal cycler (Applied Biosystems, Foster City, CA, USA) with SYBR Green PCR Master Mix (Thermo Fisher Scientific) and the PCR primers that are listed in Table 2.

**Table 2:** RT-qPCR primers.

Gene	Sequence	References
Arc/Arg3.1 forward	AGCCAGGAGAATGACACCAG	Mateos et al. (2009)
Arc/Arg3.1 reverse	GGCAGCTTCAGGAGAAGAGA	
GAPDH forward	CATCAAGAAGGTGGTGAAGCA	Rao et al. (2006)
GAPDH reverse	CTGTTGAAGTCACAGGAGACA	

### Protein Production in Bacteria and GST Pulldown

GST-fused Arc or GST alone was expressed in the BL21 CodonPlus strain of *E. coli* (Agilent) and purified using glutathione Sepharose 4B (GE Healthcare, Chicago, IL, USA) according to the manufacturer’s instructions. Beads were washed and incubated for 1 h with lysate from the rat hippocampal CA region (1 mg/ml) prepared in lysis buffer (50 mM Tris-HCl [pH 7.4], 150 mM NaCl, 1 mM ethylenediaminetetraacetic acid, 0.5% NP-40 supplemented with 1 mM phenylmethanesulfonyl fluoride [PMSF], and 1× Complete Protease Inhibitor Cocktail [Sigma-Aldrich]). The beads were then washed with lysis buffer, and bound proteins were eluted by boiling in Laemmli buffer. GSK3α/β binding was detected using an anti-GSK3α/β antibody. GST-fused proteins were visualized by the “stain-free” technique (Bio-Rad, Hercules, CA, USA).

### Protein Overexpression in HEK293 Cells and Protein Purification

HEK293 cells were grown under standard conditions to 70% confluence and transfected with BIO-Arc or BIO-βGal together with BirA plasmid using jetPEI (Polyplus-transfection, Illkirch, France). Forty-six hours later, the cells were treated with the reversible GSK3α/β inhibitor CH98 (1 μM) for 2 h to inhibit GSK3α/β-dependent protein phosphorylation within cells, harvested by scraping in PBS with 100 mM NaF, and centrifuged at 1000× *g* at 4°C for 10 min. The cells were then lysed in buffer that contained 20 mM Tris (pH 8.0), 150 mM KCl, 1% Triton, and protease and phosphatase inhibitors (Sigma-Aldrich) and centrifuged at 15,000× *g* for 10 min at 4°C. The supernatant was collected, loaded on streptavidin-coated beads (Dynabeads M280, Thermo Fisher Scientific), and rotated for 3 h at 4°C, followed by extensive washes in cell lysis buffer with 500 mM KCl and 2% Triton X-100. Finally, biotinylated protein-containing beads were resuspended in appropriate buffer, depending on the further application (i.e., avi-tag pulldown, *in vitro* kinase assay, and MS).

### Avi-tag Pulldown

Beads with captured Arc or β-Gal (prepared as described above) were washed twice in pulldown buffer (20 mM HEPES [pH 7.3], 150 mM KCl, 0.25% NP-40, and protease and phosphatase inhibitors) and rotated with the homogenate of the mouse hippocampus that was prepared in the same buffer for 4 h at 4°C, followed by four mild washes in pulldown buffer and boiling in 2× Laemmli buffer. The level of GSK3α/β that bound to biotinylated proteins was evaluated by immunoblotting.

### Avi-tag Co-Precipitation

HEK293 cells were co-transfected with BIO-Arc, BirA and wildtype or inactive forms of GSK3α or GSK3β. Twenty hours posttransfection, the growth medium was replaced with DMEM that contained 1000 mg/ml glucose and 10% FBS for 4 h, followed by cell lysis in avi-tag pulldown buffer and centrifugation at 15,000× *g* for 10 min at 4°C. Arc protein complexes were captured on streptavidin-coated beads, washed four times in the same buffer, and denatured. Arc-GSK3α/β complexes were then analyzed by Western blot.

### Western Blot

Proteins that were denatured in Laemmli buffer were separated by sodium dodecyl sulfate polyacrylamide gel electrophoresis (SDS-PAGE) and transferred to nitrocellulose membranes. The membranes were blocked in 5% nonfat milk in TBS-T buffer and incubated with primary antibody overnight at 4°C. The membranes were then washed and incubated with secondary HRP-conjugated antibodies or LI-COR fluorescently labeled secondary antibodies, followed by ECL detection or analysis with the LI-COR Odyssey Imaging System (LI-COR Biosciences). For the detection of biotinylated proteins, Western blot membranes were incubated overnight with IRDye Streptavidin 800CW at 1:1000 dilution (LI-COR Biosciences) and analyzed with the LI-COR Odyssey Imaging System.

### *In Vitro* Kinase Assay and Autoradiography

Beads with captured Arc were prepared as described above, rinsed twice in kinase assay buffer (50 mM HEPES [pH 7.3] and 15 mM MgCl_2_), and incubated with or without recombinant human GSK3α or GSK3β (2 ng/μl, Merck Millipore) in the presence of 2.5 μCi of [γ-P^33^] ATP (Hartmann Analytic GmbH, Braunschweig, Germany) and 100 μM cold ATP for 30 min at 30°C. The beads were then washed twice with the buffer that was used for HEK293 cell lysis and then boiled in 2× Laemmli buffer. The samples were resolved by SDS-PAGE. The gel was stained with Coomassie Brilliant Blue, dried, and autoradiographed. The radioactive signal was detected using a Typhoon Trio+ Phosphorimager (GE Healthcare).

### MS/MS Analysis of Protein Ubiquitination

To identify posttranslational modifications of Arc protein, liquid chromatography coupled with MS was performed as described previously (Graczyk et al., 2011; Zaremba-Czogalla et al., 2011), with modifications. For the identification of GSK3β-dependent ubiquitination, Arc protein that was captured on streptavidin-coated beads was reduced with 5 mM Tris(2-carboxyethyl)phosphine (TCEP, RT, 1 h), methylthiolated with 10 mM methyl metanethiosulfonate (MMTS, RT, 10 min) to block free thiols, and digested with 10 ng/μl trypsin (Promega, Fitchburg, WI, USA) overnight at 37°C. The peptide mixture was concentrated and desalted on an RP-C18 pre-column (nanoACQUITY Symmetry C18, Waters, Milford, MA, USA), and further peptide separation was achieved using a nanoACQUITY nano-Ultra Performance Liquid Chromatography (UPLC) RP-C18 column (BEH130 C18 column, 75 μm inner diameter, 250 mm length, Waters) with an acetonitrile gradient (5%–35% AcN for 45 min) in the presence of 0.05% formic acid at a flow rate of 150 nl/min. The column outlet was directly coupled to the electrospray ionization source of an Orbitrap Velos type mass spectrometer (Thermo Fisher Scientific), working in the regime of data dependent MS to MS/MS switch with higher energy collisional dissociation (HCD) type of peptide fragmentation. An electrospray voltage of 2 kV was used. Raw data files were pre-processed using Mascot Distiller 2.4.2.0 software (MatrixScience). The peptide masses and fragmentation spectra were matched to the Swiss-Prot database (548586 sequences/195452300 residues) with a *Rattus* filter (7937 sequences) using the Mascot search engine (Mascot Daemon v. 2.4.0, Mascot Server v. 2.4.1, MatrixScience) and the following search parameters: enzyme specificity set to trypsin, protein mass left unrestricted, and mass values as monoisotopic with one missed cleavage allowed. The oxidation of methionine, the methyltiolation of cystein, the phosphorylation of serine, threonine, and trypsin, and ubiquitination were set as modifications. Peptides with a Mascot score that exceeded the threshold value (corresponding to <1% false positive rate, calculated by the Mascot procedure) were considered to be positively identified.

### MS/MS Analysis of Protein Phosphorylation

To identify phosphorylated residues within the Arc sequence, rat Arc protein was overexpressed in HEK293 cells, purified as described above, denatured, and subjected to SDS-PAGE. The gel was stained with Coomassie Brilliant Blue, and bands that corresponded to Arc were excised. The samples were then subjected to “in-gel digestion” with trypsin, during which the proteins were reduced with 100 mM DTT (30 min at 56°C), alkylated with 0.5 M iodoacetamide (45 min in a darkroom at room temperature), and digested with trypsin. The resulting peptides were eluted from the gel with 0.1% trifluoroacetic acid (TFA). To improve Arc protein digestion, the trypsin-digested samples were acidified and additionally digested by pepsin (Immobilized Pepsine, Thermo Fisher Scientific) for 1 h at 24°C. Digested peptides were mixed with loading buffer (80% acetonitrile, 5% TFA, and 1 M phthalic acid) and incubated with an equilibrated MagReSyn Ti-IMAC microsphere pellet (ReSyn Biosciences, Edenvale, South Africa) for 20 min. The supernatant was then removed, and microspheres with chelated titanium ions were washed with loading buffer and next with wash buffer (80% acetonitrile and 1% TFA). Phospho-peptides were eluted with water that was alkalized with ammonia to pH 10.5 and immediately acidified with 50% TFA after elution. The obtained peptides were applied to an RP-C18 pre-column as described above. The acquired raw data were processed by the Mascot distiller, followed by a database search with the Mascot program against the Users database. The search parameters for precursor and product ion mass tolerance were 30 ppm and 0.1 Da, respectively, with no enzyme specificity, fixed modifications through cysteine carbamidomethylation, and the following variable modifications: methionine oxidation, and serine, threonine, and tyrosine phosphorylation. Peptides that were identified in the Mascot search as phosphorylated were subjected to the confirmation procedure based on visual inspection of the fragmentation spectra that corresponded to the modified (and unmodified, when detected) peptide and identification of a significant fraction of expected product ions.

### Immunocytochemistry, Image Acquisition and Analysis

For endogenous Arc detection, hippocampal neurons that were grown on coverslips were fixed with methanol at −20°C for 2 min, followed by fixation in 4% paraformaldehyde (PFA)/4% sucrose in PBS for 10 min at room temperature. The fixed cells were then incubated with blocking solution (10% normal goat serum and 0.1% Triton X-100 in PBS) for 1 h at 37°C, incubated with rabbit anti-Arc antibody in GDB buffer (0.2% gelatin, 0.8 M NaCl, 0.5% Triton X-100, and 30 mM phosphate buffer, pH 7.4; Jaworski et al., 2005) overnight at 4°C, and incubated with secondary antibody for 1 h at room temperature in the same buffer. For all of the other stainings, the cells were fixed with PFA as described above and incubated with appropriate antibody in GDB. To evaluate protein expression, Z-stack images were acquired with a Zeiss NLO710 confocal microscope at 40× magnification and 512 × 512 pixel resolution, and each Z-stack image was averaged twice. The microscope settings were kept constant for the entire experiment. To reveal endogenous Arc expression, single Z-stack images were compressed to obtain the maximum intensity projection. To measure the fluorescence intensity of HA-tagged Arc and mCherry in single Z-stack images, MetaMorph image analysis software (Molecular Devices, Sunnyvale, CA, USA) was used. The HA fluorescence intensity that was measured from single Z-stacks was then divided by the mCherry fluorescence intensity from the same Z-stack. The obtained values were summed, yielding HA-Arc expression that was normalized to mCherry expression. To analyze dendritic protrusions properties, neurons were co-transfected with β-actin-GFP, which allowed the visualization of cell morphology. To enhance the GFP signal, staining with anti-GFP antibody was performed. HA-tagged Arc expression was detected as above, when appropriate. Images were acquired with a Zeiss NLO800 Airyscan confocal microscope and 63× objective. Confocal cell images were obtained at 1024 × 1024 pixel resolution. Each image was a z-series of images, which were averaged twice per line. Z-stack images were compressed to obtain maximum intensity projections. The width of dendritic protrusions on secondary and tertiary dendrites was measured using MetaMorph software. When protrusion width was ≤0.4 μm, protrusion was classified as filopodium. All other protrusions were classified as dendritic spines and divided into three categories: spines without distinct neck were classified as stubby, spines with neck and head diameter equal or above medium spine width calculated for all experiments (0.78 μm) were considered as mushroom spines and spines with neck and head diameter <0.78 μm were classified as thin ones. Finally, percentage of filopodia and each class of dendritic spines on dendrite segment was calculated.

### Statistical Analysis

The data are expressed as mean ± standard error of the mean (SEM). The statistical analysis was performed using Prism 5 software (GraphPad, La Jolla, CA, USA). When multiple comparisons between experimental groups were performed One-way ANOVA followed by Bonferroni *post hoc* test was used. When only two groups (treatment vs. respective control) *t*-test or Mann-Whitney test (for data lacking normal distribution) was applied. In case when control = 1 for each experiment One-sample *t*-test was used for comparison of each experimental variant to control.

## Results

### GSK3α/β Inhibition Upregulates NMDAR-Dependent Arc Expression and Alters Dendritic Spine Morphology

Although GSK3α and GSKβ play a vital role in the plasticity of excitatory synapses (Peineau et al., 2007, 2009; Cymerman et al., 2015), very few relevant effectors have been identified. One important cellular function of GSK3α/β is to mark proteins for degradation (Xu et al., 2009). Therefore, we examined whether GSK3α/β inhibition affects the levels of Arc, a synaptic plasticity protein with very fast turnover, the expression of which is quickly terminated by the ubiquitin-proteasome system (Rao et al., 2006; Greer et al., 2010; Soulé et al., 2012; Bateup et al., 2013; Mabb et al., 2014). Arc protein levels in unstimulated 15 day *in vitro* (DIV) cortical neurons were very low and increased upon 4 h stimulation with 10 μM NMDA (Figures 1A,B). This result was consistent with previous reports that showed that NMDARs are crucial for the induction of Arc expression in neurons (Lyford et al., 1995; Rao et al., 2006; Bloomer et al., 2008; Kawashima et al., 2009; Panja et al., 2009; Bateup et al., 2013). The adenosine triphosphate (ATP)-competitive GSK3α/β inhibitors BIO and CH98 at concentrations that efficiently blocked β-catenin degradation (Figure 1C) augmented the effect of NMDA on Arc expression (Figures 1A,B). GSK3α/β inhibition also promoted Arc protein accumulation in hippocampal neurons that were stimulated with NMDA, revealed by immunocytochemical staining. Arc resided primarily in dendrites, but it was also localized to the cell nucleus, especially in neurons with abundant Arc expression (Figure 1D). The specificity of Arc IF was confirmed under conditions of Arc knockdown (Figure 1E). Next, we investigated whether GSK3α/β inhibition influences Arc mRNA levels in NMDA-stimulated cortical neurons. We found no difference between neurons that were exposed to NMDA alone and neurons that were exposed to both NMDA and GSK3α/β inhibitors (Figure 1F). Thus we concluded that GSK3α/β regulate Arc protein expression upon NMDAR activation without affecting Arc mRNA levels.

Arc overexpression results in a higher proportion of thin dendritic spines and decrease in medium spine width (Peebles et al., 2010). First, we investigated the effect of prolonged NMDAR stimulation and GSK3α/β inhibition, shown above to increase Arc expression, on dendritic protrusion width. A tendency toward a decrease in protrusion width was observed in cells that were treated with CH98 alone (4.3% reduction). Treatment with BIO alone and NMDA alone decreased protrusion width by 9.5% and 10.1%, respectively (Figures 2A,B). When neurons were exposed to both NMDA and CH98, a 21.7% reduction of protrusion width was observed compared with vehicle-treated cells. The combination of BIO and NMDA led to 30% decrease while comparing to control neurons. These results demonstrate the synergistic actions of GSK3α/β inhibitors and NMDA on average width of dendritic protrusion (Figures 2A,B). Analysis of cumulative distribution of protrusion width further confirmed their thinning Figure 2C). Next, dendritic protrusions were classified as filopodia, mushroom, stubby and thin spines, based on their width and morphology (as described in “Materials and Methods” Section) and the percentage of each protrusion type was calculated. The concomitant inhibition of GSK3α/β and NMDAR stimulation potently increased percentage of filopodia and reduced the contribution of mushroom spines, while proportion of stubby and thin spines didn’t vary significantly between conditions (Figure 2D).

### GSK3α/β Promote Arc Degradation in NMDA-Treated Neurons

Multiple GSK3α/β-phosphorylated proteins are ubiquitinated and subsequently directed to proteasomal degradation (Xu et al., 2009). To investigate whether Arc protein stability is regulated by GSK3α/β, cortical neurons were treated with NMDA alone or in a combination with CH98 and then exposed to the translation blocker anisomycin (Figure 3A). Arc protein decay was slower upon GSK3α/β inhibition, indicating that GSK3α/β control Arc protein stability in NMDA-treated neurons (Figures 3B,C). A similar effect was observed when protein synthesis was blocked with another translation inhibitor, cycloheximide (Supplementary Figure S1).

Next, we sought to determine the lysine residue(s) of Arc that were ubiquitinated in a GSK3α/β-dependent manner. We performed mass spectrometry (MS) analysis of rat Arc co-expressed in human embryonic kidney 293 (HEK293) cells without and with the constitutively active form of GSK3β (GSK3β S9A). Cells were treated for 1.5 h with the proteasome inhibitor MG-132 to preserve ubiquitinated Arc from proteasome-dependent degradation. MS identified Arc lysine 136 (K136) as a residue that is ubiquitinated in a GSK3β-dependent manner, in which the ubiquitination of lysine 136 (K136) was detected only upon co-expression with GSK3β S9A (Figure 3D). This ubiquitination appeared to be conserved across species because the same modification was identified in human Arc co-expressed with GSK3β S9A (data not shown).

Subsequently, we evaluated the contribution of Arc lysine 136 ubiquitination to Arc degradation. We employed lentivirus-based synaptic activity-responsive element (SARE)-Arc constructs, in which Arc expression is driven by the SARE-ArcMin promoter (Kawashima et al., 2009; Figure 3E). This allowed the construct to respond to neuronal stimulation (e.g., NMDA treatment, Figure 3F). The Arc mutant in which lysine 136 was substituted with arginine (Arc K136R) decayed more slowly in NMDA-stimulated cortical neurons, thus demonstrating the importance of the newly identified ubiquitination site for Arc degradation (Figures 3F,G). To further confirm that lysine 136 ubiquitination depends on GSK3β, we compared the expression of wildtype SARE-Arc and the K136R mutant upon NMDAR stimulation in neurons that were co-transfected with an empty vector (β-actin-16pl) or with the GSK3β S9A construct (Figures 3H,I). The overexpression of constitutively active GSK3β prevented the NMDAR-dependent increase in wildtype Arc but not Arc K136R mutant levels. These results indicate that the ubiquitination of lysine 136 in Arc is required for GSK3β-dependent Arc decay.

### GSK3α/β-Mediated Arc Phosphorylation Regulates its Stability

**Figure 4:**
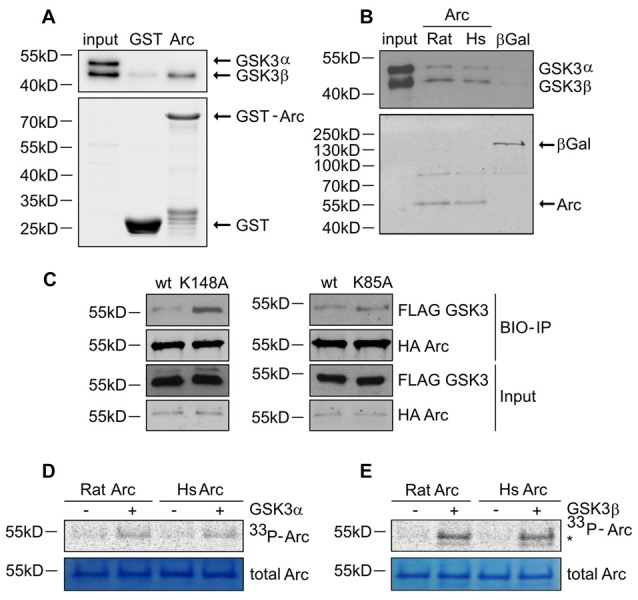
GSK3α/β binds and phosphorylates Arc protein. **(A)** GSK3β directly associates with Arc protein. Purified GST-Arc protein of rat origin was incubated with protein lysate from rat hippocampus. GSK3α/β binding was determined by Western blot. **(B)** GSK3α/β may directly associate with Arc protein in cells. Rat and human Arc or β-Gal proteins that were overexpressed in HEK293 cells were captured on streptavidin-coated beads and incubated with mouse hippocampal lysate. Bait-kinase binding was estimated by probing the blot with anti-GSK3α/β antibody. Bait (e.g., biotinylated Arc or β-Gal) was detected using fluorescently labeled streptavidin. **(C)** Kinase dead (KD) mutants of GSK3α (K148A) and GSK3β (K85A) display greater affinity to rat Arc protein than wildtype kinases. HA-tagged BIO- Arc protein was overexpressed in HEK293 cells together with a wildtype or KD form of FLAG-GSK3α (K148A) or a wildtype or KD form of FLAG-GSK3β (K85A) for 20 h. Cell lysates were then harvested and immediately subjected to avi-tag co-precipitation. Blots were probed with anti-FLAG antibody to detect overexpressed GSK3α/β proteins and probed with anti-HA antibody to detect recombinant Arc. **(D,E)** Rat and human Arc proteins are phosphorylated *in vitro* by recombinant GSK3α and GSK3β. The figures show representative results from the *in vitro* kinase assay. Arc proteins that were overexpressed in HEK293 cells were isolated and incubated with or without recombinant GSK3α or GSK3β. Arc phosphorylation was detected by autoradiography. The level of total Arc was estimated with Coomassie Brilliant Blue staining. The phosphorylated GSK3β band is visible beneath the Arc band (indicated by asterisk [*]).

**Figure 5:**
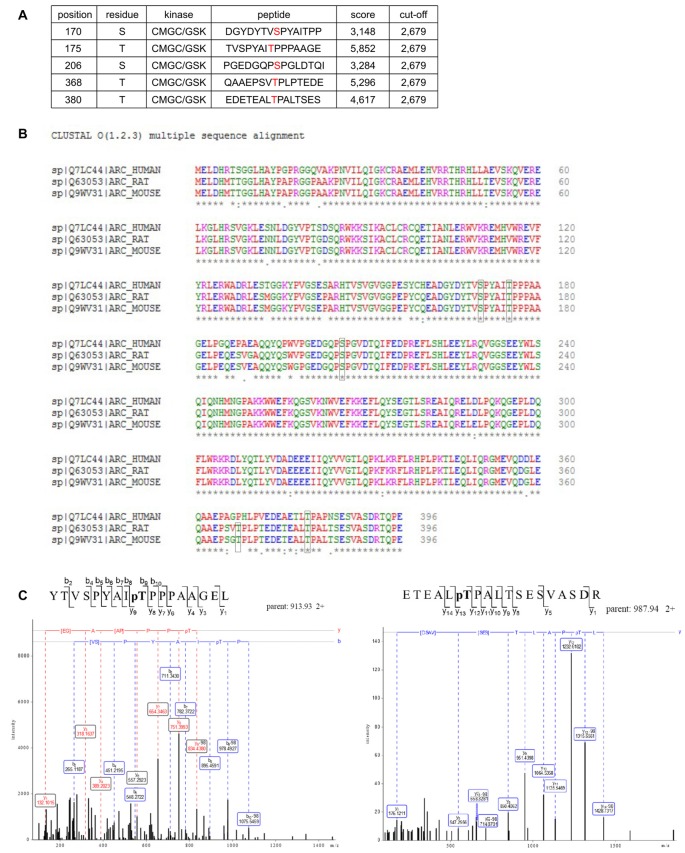
Potential GSK3 phosphorylation sites are present in Arc sequence. **(A)** Bioinformatic prediction of potential GSK3β phosphorylation sites in the rat Arc sequence based on Group Based Predictor Phosphorylation System 2.1 (GPS 2.1). The potential GSK3β-targeted residues of Arc are listed in the table. **(B)** Sequence alignment of human, rat, and mouse Arc amino acid sequences. Residues that are potentially targeted by GSK3β are marked with black boxes. **(C)** Mass spectrometry (MS)/MS analysis indicates phosphorylated residues within Arc protein. The figure shows exemplary phosphopeptides that were identified by phospho-MS analysis of rat Arc, overexpressed in HEK293 cells. Note the neutral loss (−98 Da) visible on threonine 175 (left panel) and threonine 380 (right panel), indicating the phosphorylation of these residues in living cells.

Arc in neurons is ubiquitinated and subsequently degraded in a GSK3α/β-dependent manner. Therefore, we evaluated whether GSK3α and GSK3β bind directly to and phosphorylate Arc protein. GSK3β was pulled down on GST-tagged bacterially expressed Arc (Figure 4A). Similarly, both human and rat Arc protein that were overexpressed and isolated from HEK293 cells were observed to bind GSK3α/β (Figure 4B). Within the cell, active kinase and substrate form very transient complexes prior to substrate phosphorylation (Belozerov et al., 2014). We hypothesized that the binding of an enzymatically inactive kinase mutant to Arc should be stronger than the binding of wildtype kinase. Indeed, Arc bound enzymatically inactive forms of GSK3α and GSK3β (K148A and K85A mutants, respectively) more efficiently than wildtype enzymes (Figure 4C). These findings suggested that indeed GSK3α/β might serve as an Arc kinase. We then demonstrated this directly by performing *in vitro* kinase assays and found that both GSK3β and GSK3α phosphorylated rat and human Arc (Figures 4D,E). To identify potential residues of Arc that are phosphorylated by GSK3α/β, we utilized Group Based Predictor System v. 2.1 (GPS2.1), a bioinformatic tool that allows the prediction of phosphorylation sites for selected kinases in a given protein sequence (Xue et al., 2011). The prediction returned five hits, including serines or threonines followed by proline: serine 170, threonine 175, serine S206, threonine 368 and threonine 380 (Figure 5A). Serine 170, threonine 175, serine S206 and threonine 380 are conserved from rat and mouse to human Arc protein (Figure 5B). The tandem MS analysis of Arc overexpressed in HEK293 cells (pretreated with CH98) and subsequently phosphorylated by GSK3β *in vitro*, upon enrichment on titanium dioxide allowed the identification of Arc-derived phosphopeptides that encompassed serine 170, threonine 175, and threonine 380 (Figure 5C). It should be noted however, that those sites were identified also in Arc protein purified from HEK293 cells pretreated with CH98 and not phosphorylated *in vitro*, inferring that these sites could be phosphorylated by GSK3α/β and/or by other kinases (e.g., as prephosphorylation sites for GSK3). Serine 206 has also been recently confirmed to be phosphorylated by another kinase *in vitro* and *in vivo* (Nikolaienko et al., submitted).

**Figure 6:**
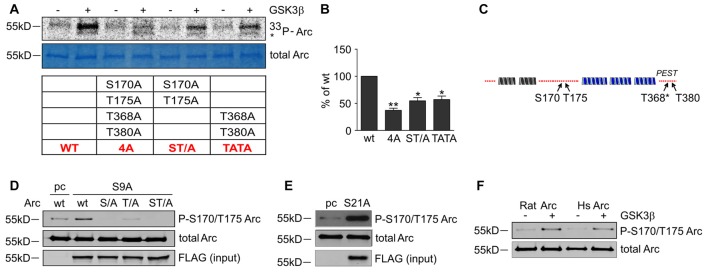
GSK3-dependent Arc phosphorylation requires presence of serine 170 and threonines 175, 368 and 380 of Arc. **(A)** Predicted and MS-indicated residues of Arc required for GSK3-mediated phosphorylation *in vitro*, demonstrated by the *in vitro* kinase assay. The figure shows representative results of the *in vitro* kinase assay. The S170, T175, T368 and T380 residues were substituted with alanines, and quadruple (all four residues mutated [4A]) or double (S170A/T175A and T368A/T380A) mutants were subjected to *in vitro* phosphorylation by GSK3β, as indicated in the table below. **(B)** Quantitative analysis of kinase assay results. The data represent the levels of Arc phosphorylation in the variants, where GSK3β was present. The optical density of the autoradiography bands was normalized to the optical density of Coomassie Blue staining bands, and the obtained value for each mutant was compared with GSK3β-phosphorylated wildtype Arc levels. The data are expressed as means ± SEM from three independent assays. *p < 0.05, **p < 0.01 (One sample *t*-test). **(C)** Schematic representation of Arc protein. Blue and gray helices represent structured protein domains, the lines of red dots symbolize regions without defined secondary structure (disordered regions). Residues that are critical for GSK3α/β-mediated phosphorylation are indicated with arrows. **(D)** GSK3β-mediated Arc phosphorylation at S170/T175 was confirmed with an antibody that was developed against a peptide that encompassed phosphorylated S170 and T175 of Arc. The figure shows the Western blot analysis of phosphorylated Arc expression. HEK293 cells were transfected with wildtype BIO-Arc together with pcDNA3 vector (pc) or wt BIO-Arc, S170A (S/A), T175A (T/A) or S170A/T175A (ST/A) BIO-Arc together with FLAG GSK3β S9A. Twenty-four hours posttransfection, the cells were harvested, and Arc protein was isolated. Blots were probed with anti-P-S170/T175 Arc antibody and rabbit anti-Arc antibody. Additionally, FLAG GSK3β S9A was detected in input fraction. Note the lack of a P-S170/T175 Arc band in S170A and S170A/T175A mutant lanes and signal reduction in T175A lane. **(E)** GSK3α also upregulates phosphorylation of Arc S170/T175. The figure shows the Western blot analysis of phosphorylated Arc expression. HEK293 cells were transfected with wildtype BIO-Arc together with pcDNA3 vector (pc) or GSK3α S21A. Twenty-four hours posttransfection, the cells were harvested, and Arc protein was isolated. Blots were probed with anti-P-S170/T175 Arc antibody and rabbit anti-Arc antibody. **(F)** Western blot showing rat and human Arc phosphorylation by GSK3β in the *in vitro* kinase assay. The assay was performed as in **(B)**, and the blot was probed with anti-P-S170/T175 Arc antibody and rabbit anti-Arc antibody.

To determine whether the predicted residues in Arc protein are important for GSK3β-mediated phosphorylation, we generated unphosphorylatable mutants of Arc by substituting S170, T175, T368 and T380 with alanines and performed an *in vitro* kinase assay. The levels of GSK3β-mediated phosphorylation of the double Arc mutants S170A/T175A and T368A/T380A were reduced by 45% and 43%, respectively, compared with wildtype Arc phosphorylation. The phosphorylation of the quadruple Arc mutant (later referred to as 4A) was 63% lower compared with wildtype Arc (Figures 6A,B). The bioinformatic analysis of Arc sequence followed by biochemical and biophysical characterization of Arc protein implied the presence of two protein domains with defined structure (Myrum et al., 2015; Zhang et al., 2015), separated by more disordered central region (Myrum et al., 2015). Putative GSK3α/β-targeted residues S170 and T175 are localized to this central disordered region of Arc, and T368 and T380 are part of the putative PEST sequence (aa 351–392) that is located at the Arc C-terminus (Rao et al., 2006; Figure 6C). We next employed an anti-phospho S170/T175 Arc antibody to confirm the GSK3β-mediated phosphorylation of these residues. The co-expression of rat Arc together with GSK3β S9A and GSK3α S21A in HEK293 cells potently increased S170/T175 phosphorylation (Figures 6D,E). Anti-P-Arc antibody did not recognize Arc when S170 is mutated to alanine, however single T175A mutant is still recognized by anti-P-Arc antibody (Figure 6D). This result suggests that both phosphorylated residues are detected by anti-P-Arc antibody, although with slightly different efficiency and in particular confirms that GSK3β targets S170 despite the lack of detection of phosphorylated S170 by MS analysis. Arc proteins of rat and human origin that were phosphorylated *in vitro* by GSK3β (as in Figure 4D) presented a much higher level of S170/T175 phosphorylation compared with control conditions (i.e., incubation without kinase; Figure 6F).

**Figure 7:**
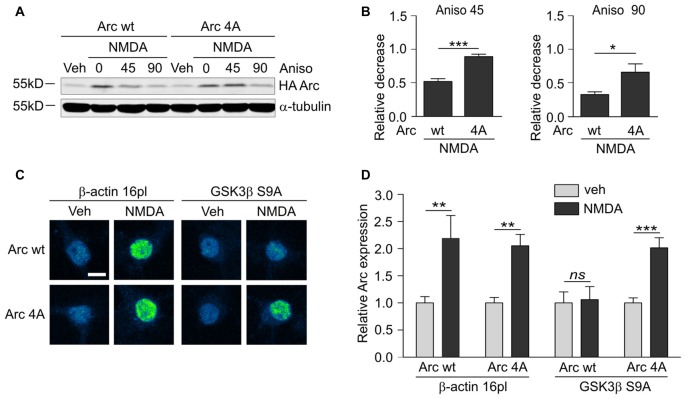
GSK3α/β-mediated phosphorylation leads to Arc degradation in neurons. **(A)** Quadruple (4A) mutant of Arc is more stable than wildtype protein in NMDA-stimulated neurons. The figure shows the Western blot analysis of HA-tagged Arc overexpressed in cortical neurons. Cortical neurons were transduced with lentiviral vectors that carried wildtype SARE-Arc or SARE-Arc4A. Twenty-one hours posttransduction, the cells were treated with NMDA for 4 h, and anisomycin was added for the times indicated. **(B)** Quantification of Western blot results from **(A)**. Next, relative decrease in HA-Arc expression was calculated as a ratio of HA-Arc level at a given time point (45 or 90 min of anisomycin treatment) to HA-Arc level at the “time 0”, when anisomycin was added to neurons pretreated with NMDA. Decrease in Arc and Arc 4A levels was compared separately for 45 or 90 min of anisomycin treatment and the difference was evaluated with *t*-test. The data are expressed as mean ± SEM (*n* = 5 independent cultures). *p < 0.05, **p < 0.01 (*t*-test). **(C)** Unphosphorylatable mutant of Arc is insensitive to GSK3β- mediated degradation. The figure shows representative confocal images of HA-tagged Arc expression in hippocampal neurons that were co-transfected with β-actin 16pl vector or GSK3β S9A and wildtype SARE-Arc or the 4A mutant together with SARE-mCherry plasmid for 20 h and stimulated with NMDA for 4 h. Images were transformed to “blue to yellow” pseudo color mode to visualize differences in immunofluorescence intensity. Scale bar = 10 μm. **(D)** Quantitative analysis of Arc expression. HA immunofluorescence was measured and normalized to mCherry fluorescence. For each kind of transfection, the normalized expression of HA-tagged Arc in NMDA-treated neurons was compared with Arc expression in control cells. Sixty to seventy cells per condition from three independent cultures were analyzed. **p < 0.01, ***p < 0.001; ns, not significant (Mann-Whitney test).

Finally, we determined whether the identified GSK3β-phosphorylated residues of Arc are important for protein degradation in NMDA-stimulated neurons. We created a lentiviral vector that carried the 4A mutant of SARE-Arc and evaluated its rate of degradation compared with wildtype SARE-Arc. NMDA-stimulated cortical neurons that were transduced with wildtype SARE-Arc or SARE-Arc 4A were harvested at time “0” or treated with anisomycin for 45 or 90 min. The rate of Arc degradation was then evaluated by Western blot. The Arc 4A mutant that was resistant to GSK3β-mediated phosphorylation decayed much slower than wildtype protein (Figures 7A,B), showing that phosphorylation by GSK3β regulates Arc protein degradation in neurons. We then compared the levels of the NMDAR-dependent induction of wildtype Arc vs. Arc 4A expression in neurons that were co-transfected with an empty vector (β-actin-16pl) or GSK3β S9A. The NMDAR-dependent increase in wildtype Arc expression was abolished by constitutively active GSK3β, whereas the expression of the unphosphorylatable Arc mutant was unaffected (Figures 7C,D). These results indicate that the identified residues within Arc are critical for GSK3β-mediated Arc degradation.

### GSK3α/β-Mediated Arc Protein Degradation Contributes to the Structural Plasticity of Dendritic Spines

**Figure 8:**
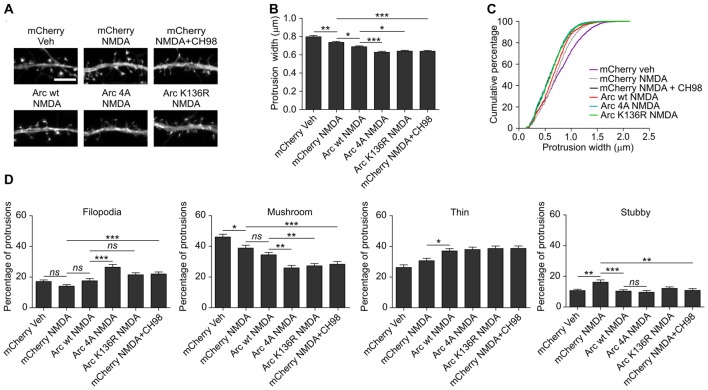
The overexpression of K136R and 4A Arc mutants enhances reduction of dendritic protrusion width and alterations in dendritic spine morphology. Hippocampal neurons were transfected with SARE-mCherry, wildtype SARE-Arc, SARE-Arc K136R, or SARE-Arc 4A together with β-actin-GFP to visualize cell morphology and stimulated with 10 μM NMDA for 4 h. CH98 (1 μM) was added where indicated. **(A)** Representative images of GFP fluorescence in dendrite fragments of neurons that were transfected and treated as indicated. Scale bar = 5 μm. **(B)** Quantitative analysis of dendritic protrusion width. Measurements were averaged per dendrite segment. Two dendrite segments per cell from 20 to 27 cells from three independent cultures were analyzed (6–9 cells per culture). The data are expressed as the mean protrusion width ± SEM. *p < 0.05, **p < 0.01, ***p < 0.001 (one-way ANOVA with Bonferroni correction for multiple comparisons). **(C)** Cumulative percentage plot of spine width for neurons from **(B)**. Calculations were done for 1700–2000 protrusions per condition. **(D)** Analysis of dendritic protrusion categories for neurons from **(B)**. Categorization was done for dendrite segment. Two dendrites per cell were analyzed. Data are expressed as mean percentage of protrusions ± SEM. *p < 0.05, **p < 0.01, ***p < 0.001 (one-way ANOVA with Bonferroni correction for multiple comparisons).

As shown above, conditions that increased Arc expression and stability (i.e., simultaneous treatment with NMDA and GSK3α/β inhibitors) resulted in a reduction of dendritic protrusion width and subsequent increase in a proportion of filopodia and decreased percentage of mushroom spines (Figure 2). The overexpression of wildtype Arc enhanced the NMDAR-dependent decrease in protrusion width (Figures 8A,B). Observed protrusion shrinkage was further augmented by the introduction of ubiquitination-resistant (K136R) and unphosphorylatable (4A) mutants of Arc, both decreasing protrusion width to the similar extent as observed in neurons that were exposed to NMDA and CH98 (Figures 8A,B). Also the cumulative distribution plot shows a similar shift toward thinner protrusions in neurons transfected with Arc mutants and mCherry transfected cells treated with NMDA and CH98 (Figure 8C). Next, we determined how wildtype Arc or Arc mutants affect distinct classes of dendritic protrusions. We found decreased proportion of stubby spines and increased proportion of thin spines in NMDA-treated neurons overexpressing wildtype Arc while comparing to NMDA-treated cells transfected with SARE-mCherry (Figure 8D). The comparison of dendritic protrusions distribution in wildtype Arc overexpressing neurons and neurons overexpressing ubiquitination-resistant (K136R) and unphosphorylatable (4A) mutants of Arc treated with NMDA revealed higher percentage of filopodia in neurons transfected with 4A mutant of Arc and decreased percentage of mushroom spines in cells overexpressing 4A and K136R Arc mutants (Figure 8D). Functionally, the increase in Arc stability translates into a more pronounced reduction of dendritic protrusion width and promotes loss of mushroom spines. These results underscore the importance of GSK3α/β-mediated Arc decay for the structural plasticity of dendritic spines.

## Discussion

In the present study, we found that Arc, a protein that is crucial for synaptic function, is phosphorylated and degraded in a GSK3α/β-dependent manner upon prolonged NMDA receptor stimulation. We further elucidated the effects of this degradation on dendritic spine morphology.

### GSK3α and GSK3β Phosphorylate Arc and Direct it Toward Degradation

The present study provided evidence that GSK3α/β directly phosphorylates Arc, resulting in its subsequent degradation. Our findings demonstrate a novel role of Arc phosphorylation. The mechanisms of Arc transcription and translation have been studied quite intensively. Arc also undergoes variety of posttranslational modifications, e.g., ubiquitination, SUMOylation and phosphorylation. Thus far and as discussed below, Arc ubiquitination was most extensively studied. Arc mono-SUMOylation correlated with interaction of Arc with drebrin A, an F-actin binding protein, during LTP consolidation (Nair et al., 2017). The role of Arc phosphorylation, however, has remained unknown.

Previous studies reported that Arc is phosphorylated at threonine 278 and tyrosine 274, but the functions of these modifications and respective kinases were not analyzed (Trinidad et al., 2012; Palacios-Moreno et al., 2015). In this study, the phosphorylation of two residues, namely T175 and T380 in HEK 293 cells, was identified by MS (Figure 5C). However, bioinformatic analysis suggested existence of several more phosphorylation sites in Arc, some of them being potential GSK3α/β substrates (e.g., S170, T368; Figure 5A). Subsequent experiments in HEK293 cells strongly suggested that Arc S170 indeed can be also phosphorylated. But *in vitro* kinase assays showed that only combined substitution of S170, T175, T368 and T380 with alanines potently prevented GSK3β-dependent Arc phosphorylation. Therefore 4A mutant was used for subsequent functional studies. Nevertheless a question is why S170 and T368 were not found by our MS analysis. It is possible that phospho-MS analysis leaves some phosphorylated residues undetected. In particular, peptides phosphorylated at multiple residues located close to each other are difficult to analyze (Dephoure et al., 2013). Also the efficiency of protease cleavage and fragmentation into ions may vary depending on structure and sequence of analyzed protein, and in turn influence the identification of phosphorylated residues. So although 4A mutant was in our opinion most reasonable choice for functional studies in neurons, further work will be required to confirm, which of these four residues are actually bona fide GSK3α/β substrates in neurons.

In contrast to Arc phosphorylation, the ubiquitination of Arc has been studied in more detail. Two E3 ligases (Triad3A and Ube3A) have been shown to ubiquitinate Arc at lysines 268 and 269 (Greer et al., 2010; Mabb et al., 2014). Lysine 136 in Arc was also shown to be ubiquitinated by Triad3A in a cell-free assay. However, the functional effect of this ubiquitination on Arc stability was not confirmed or further evaluated in neuronal cells because lysines 268 and 269 appeared to be solely responsible for Arc ubiquitination and degradation (Mabb et al., 2014). Mabb et al. (2014) found that the ubiquitination of lysines 268 and 269 was crucial for Arc degradation in neurons that were stimulated with bicuculline and 4-aminopyridine. In the present study, we showed in HEK293 cells using overexpressed Arc, that its Lys136 is also ubiquitinated *in vivo* in a GSK3α/β-dependent manner. Furthermore, we proved importance of Lys136 in Arc degradation in neurons in the context of NMDAR stimulation, which may have a different outcome with regard to the enzymatic activity of GSK3α/β compared with the stimulation conditions that were employed by Mabb et al. (2014). However, to fully prove this hypothesis future research should provide an evidence of GSK3α/β-dependent ubiquitination of Arc Lys136 in neurons in response to different types of neuronal stimulation including not only NMDA application but also more plasticity-related situations, like LTP or LTD. Yet, due to technical reasons, biochemical analysis of Arc ubiquitination proven to be technically very challenging.

GSK3α/β plays a well-known role in the phosphorylation of proteins that are destined for proteasomal degradation. To date, however, there is no evidence that GSK3α/β substrates can be subsequently ubiquitinated by the Arc E3 ubiquitin ligases Triad3A and Ube3A. The best studied E3 ubiquitin ligases that cooperate with GSK3α/β are β-Transducing repeat containing E3 ubiquitin protein ligase (β-Trcp), which mediates the ubiquitination of the canonical GSK3α/β target β-catenin and F-box and WD repeat domain containing 7 (FBXW7) protein family members, which direct multiple nuclear proteins to the proteasome (Xu et al., 2009; Lau et al., 2012) or regulate Rictor stability (Koo et al., 2015). In the case of proteins that are phosphorylated prior to ubiquitination, E3 ubiquitin ligase recognizes the phosphorylated amino acid sequence within the targeted protein, referred to as “phospho-degron” (Hunter, 2007). Analysis of Arc amino acid sequence indicated that DSGXXS/T sequence, recognized by β-Trcp upon phosphorylation of serine and threonine residues, is absent from Arc protein. E3 ligases from the FBXW7 family recognize the pS/pTP sequence that is also found in GSK3α/β-phosphorylated Arc. The role of these E3 ligases in the GSK3α/β-driven degradation of Arc needs to be investigated. Still, the possible interplay between the GSK3α/β-mediated degradation of Arc and Triad3A/Ube3A also cannot be excluded based on our data. Alternatively, the contribution of another, yet unidentified Arc E3 ubiquitin ligase should be considered. Some E3 ubiquitin ligases cooperate with GSK3α/β, but their target phospho-degrons are not known (Xu et al., 2009). Finally, protein degradation can be mediated by different E3 ubiquitin ligases in a cellular localization-dependent manner, such as in the case of β-catenin degradation (e.g., β-Trcp and TRIM33; Xue et al., 2015), and the same may apply to Arc protein since it resides both in cytoplasm and cell nucleus (Korb et al., 2013; Figures 1D,E). Interestingly, FBXW7 α and γ isoforms operate in cell nucleus (Lau et al., 2012). Therefore, they may be the candidate E3 ubiquitin ligases regulating nuclear Arc level, putatively in GSK3-dependent manner.

### GSK3α/β-Mediated Phosphorylation and Degradation of Arc Contributes to Dendritic Spine Plasticity

We found that the GSK3α/β-controlled stability of Arc affects dendritic spine morphology. The extended expression of Arc led to reduction of protrusion width, which is consistent with previous reports (Peebles et al., 2010) and the postulated role of Arc in the pruning of synapses (Okuno et al., 2012). The enhancement of Arc expression in neurons was achieved by the transient inhibition of GSK3α/β under conditions that promoted *de novo* Arc synthesis (Figure 1). The analysis of spine types distribution suggests that increased stability of Arc, whether achieved by pharmacological inhibition of GSK3α/β or mutation of residues responsible for GSK3α/β-dependent Arc ubiquitination or phosphorylation, promotes loss of mushroom spines (Figures 2D, 8D). Interestingly, whereas the effect of unphosphorylatable (4A) mutant of Arc and combined treatment with NMDA and CH98 on protrusion width, percentage of mushroom spines and filopodia is very much alike, K136R mutation of Arc affects protrusion width and spine morphology to lesser extent. However, this effect seems to be consistent with rate of Arc degradation upon different manipulations, e.g., K136R mutation does not prevent Arc degradation as efficiently as application of GSK3α/β inhibitor or mutating residues phosphorylated by GSK3α/β (Figures 3, 7). GSK3α/β was shown to regulate the structural plasticity of dendritic spines under basal conditions and upon chemical LTD induction. The persistent lack of GSK3β that was caused by gene knockout resulted in a decrease in spine head volume (Ochs et al., 2015) and a decrease in the spine length-to-width ratio (Kondratiuk et al., 2017). In the present study, 5 h incubation with the GSK3α/β inhibitor CH98 caused a tendency toward a reduction of spine width, while BIO reduced width by 9.5% (Figure 2). Our previous work showed that upon chemical LTD induction, GSK3α/β inhibition prevented the plasticity-induced decrease in spine width (Cymerman et al., 2015). In the present study, we found that GSK3α/β inhibition potentiated spine head thinning that was caused by prolonged NMDAR stimulation. GSK3α activity can promote spine shrinkage during short-term NMDAR activation leading to chemical LTD while GSK3α/β limit spine thinning when neurons are exposed to long-term NMDAR stimulation. This observation implies that GSK3α/β play a protective and homeostatic role in neurons, preventing spine pruning in the case of neuronal hyperexcitation (e.g., prolonged activation of NMDAR). In summary, to date, Arc transcription, Arc mRNA transportation and translation, and Arc protein localization have been shown to depend on the level of neuronal activity. Our data identify the novel mechanisms for curtailing Arc expression and function mediated by GSK3α/β-catalyzed phosphorylation and degradation.

## Author Contributions

AG, CRB and JJ designed the experiments and wrote the manuscript. AG, ON, MU, IAC, ES and MB performed the experiments and analyzed the data. MD contributed to the data analysis.

## Conflict of Interest Statement

The authors declare that the research was conducted in the absence of any commercial or financial relationships that could be construed as a potential conflict of interest.

## Footnotes

^1^http://sirna.wi.mit.edu/
