# Supplementary material for: GSK3α and GSK3β Phosphorylate Arc and Regulate its Degradation
Source: Front Mol Neurosci. 2017 Jun 16;10:192. doi: 10.3389/fnmol.2017.00192 (PMC5472658; doi:10.3389/fnmol.2017.00192)
Supplement: Supplementary file 1 [file Image_1.pdf]

Supplementary Material

GSK3α and GSK3β phosphorylate Arc and regulate its degradation

Agata Gozdz[1]*, Oleksii Nikolaienko[2], Malgorzata Urbanska[1, 3], Iwona A. Cymerman[1], Ewa Sitkiewicz[4], Magdalena Blazejczyk[1], Michal Dadlez[4], Clive R. Bramham[2], Jacek Jaworski[1, *]

*** Correspondence:** Agata Gozdz: agozdz@iimcb.gov.pl; Jacek Jaworski: jaworski@iimcb.gov.pl

1 Supplementary Figures

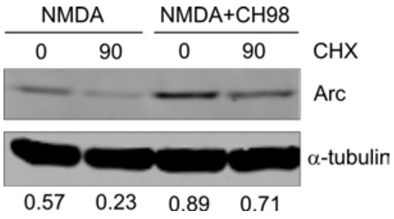

Fig S1. GSK3α/β inhibition delays Arc degradation. Cortical neurons were treated with 10 µM NMDA or 10 µM NMDA with 1 µM CH98 for 4 h and harvested (time point “0”) or treated with 10 µg/ml cycloheximide for 90 min. The ratios of Arc intensity to α-tubulin intensity are shown below.
